# Polyherbal-Mediated Synthesis of Copper Nanoparticles Using *Hygrophila auriculata* and *Leucas aspera*: Cytotoxicity, Antioxidant Effect, and Antibacterial Potential Against Healthcare-Associated Pathogens

**DOI:** 10.3390/jfb17040169

**Published:** 2026-04-01

**Authors:** Gayathri Vijayakumar, Amrutha Raja, Swathi Ganesan, Teja Sri Senthil, Jainitha Kandasamy, Prathiksha Senthil Kumaran, Senthil Kumaran Rangarajulu

**Affiliations:** 1 Department of Biotechnology, Hindustan Institute of Technology & Science, Chennai 603103, India or gayathriv2711@gmail.com (G.V.); or 24mu0950022@student.hindustanuniv.ac.in (A.R.); or 24mu0950011@student.hindustanuniv.ac.in (S.G.); or 24mu0950024@student.hindustanuniv.ac.in (T.S.S.); or 24mu0950026@student.hindustanuniv.ac.in (J.K.); 2 Department of Biotechnology, Anna University, Chennai 600025, India; pranandh2021@gmail.com; 3 Department of Biological Engineering, Konkuk University, Seoul 05029, Republic of Korea

**Keywords:** green synthesis, polyherbal extracts, copper nanoparticles, phytochemical screening, antibacterial and antioxidant activity, HepG2 cytotoxicity, DNA fragmentation

## Abstract

An eco-friendly green synthesis approach was employed to produce copper nanoparticles (CuNPs) using a polyherbal extract derived from two medicinally important plant species, *Hygrophila auriculata* (Schumach.) Heine and *Leucas aspera* (Willd.) Link. The plant extracts were initially subjected to phytochemical screening to identify bioactive constituents potentially involved in nanoparticle synthesis. The synthesized CuNPs were characterized using UV-visible spectroscopy, Fourier-transform infrared spectroscopy (FTIR), gas chromatography–mass spectrometry (GC-MS), field-emission scanning electron microscopy coupled with energy-dispersive X-ray analysis (FESEM-EDAX), X-ray diffraction (XRD), and thin-layer chromatography (TLC). UV-visible spectroscopy revealed a characteristic absorption peak at 233.6 nm. FTIR analysis indicated the presence of functional groups associated with nanoparticle reduction and stabilization, whereas FESEM imaging showed predominantly spherical particles with sizes ranging 63–68 nm. Elemental composition was confirmed using EDAX analysis. XRD analysis demonstrated polycrystalline nature of the CuNPs, with an average crystallite size of 11.5 nm. GC-MS analysis and phytochemical screening further confirmed the presence of bioactive compounds, whereas TLC analysis revealed differences in mobility between the plant extract and synthesized CuNPs. Antibacterial activity of the synthesized CuNPs was evaluated using the agar well diffusion method against clinically relevant bacterial strains, including those of *Staphylococcus aureus*, *Pseudomonas aeruginosa*, *Escherichia coli*, and *Streptococcus pyogenes*. The polyherbal-derived CuNPs produced larger inhibition zones than the individual plant extracts, particularly against multidrug-resistant pathogens such as *P. aeruginosa* and *S. aureus*. Additionally, the nanoparticles exhibited concentration-dependent antioxidant activity in the 2,2-diphenyl-1-picrylhydrazyl assay at concentrations ranging 10–50 mg/mL, with radical scavenging activity increasing from 29.9% to 76.5% and a corresponding decrease in absorbance from 0.698 to 0.234 (*p* < 0.05). Cytotoxic evaluation in HepG2 cells after 48 h of exposure demonstrated dose-dependent morphological changes and reduced cell viability. These findings suggest that polyherbal-derived CuNPs possess antibacterial, antioxidant, and cytotoxic properties with potential relevance for biomedical applications.

## 1. Introduction

Interest in eco-friendly and sustainable approaches for the synthesis of metal nanoparticles using plant-based systems rich in phytochemical constituents has increased in recent years [1,2]. Plant-mediated green synthesis has emerged as a promising alternative to conventional chemical and physical methods because plant extracts contain diverse secondary metabolites, such as flavonoids, phenolics, terpenoids, alkaloids, tannins, and glycosides, which can function as natural reducing and stabilizing agents [3]. These phytochemicals facilitate nanoparticle formation and may enhance biocompatibility and biological activity, rendering plant-derived nanoparticles particularly attractive for biomedical applications [4,5]. Various plant parts, including leaves, roots, stems, flowers, and seeds, have been successfully used for nanoparticle synthesis owing to their rich and variable phytochemical composition [6].

Medicinal plants have long been used in traditional medical systems worldwide for treating diverse ailments. Previous studies have shown that the selection of plant species and plant parts is crucial for determining nanoparticle size, morphology, stability, and biological efficacy [7,8,9]. In this context, *Hygrophila auriculata* and *Leucas aspera* were selected for the present study because of their well-documented medicinal properties and high phytochemical content, which may contribute to the synthesis and stabilization of copper nanoparticles (CuNPs) while potentially imparting additional biological functionality.

*H. auriculata* (family Acanthaceae) is widely used in Ayurvedic and Siddha medicine for treating inflammatory disorders, urinary diseases, liver ailments, wounds, and microbial infections; it is known for its antioxidant, antimicrobial, anti-inflammatory, and hepatoprotective activities [10,11]. Previous phytochemical investigations have reported the presence of flavonoids, alkaloids, tannins, and terpenoids in *H. auriculata*, highlighting its biochemical richness [12,13,14]. Similarly, *L. aspera* (family Lamiaceae) is an aromatic medicinal herb traditionally used to treat inflammation, wounds, cough, and viral infections [15]. Previous studies have shown that it contains flavonoids, phenolics, terpenoids, tannins, and essential oils and exhibits notable antibacterial, antioxidant, and anti-inflammatory properties [16,17].

CuNPs have attracted considerable attention owing to their unique physicochemical properties, including high surface area, enhanced reactivity, and relatively low cost compared with noble metal nanoparticles. These characteristics have enabled their application in fields such as electronics, textiles, agriculture, energy, and biomedicine because of their electrical, thermal, catalytic, and antimicrobial properties [18,19,20,21,22]. However, most current studies focus on the synthesis of CuNPs using extracts from a single plant species [23,24,25], whereas polyherbal green synthesis approaches remain relatively unexplored. Although *H. auriculata* and *L. aspera* contain similar classes of phytochemicals based on preliminary screening, variations in the specific composition and relative abundance of these compounds may exist between the two species. Therefore, a combined extract was investigated to provide a broadened phytochemical environment for CuNP synthesis without presuming an inherent synergistic effect. Additionally, although antimicrobial and cytotoxic activities of plant-mediated nanoparticles are frequently reported, detailed biological validation and mechanistic investigations, such as DNA fragmentation analysis to support anticancer potential, are not always thoroughly conducted [23].

In the present study, CuNPs were synthesized using a polyherbal extract of *H. auriculata* and *L. aspera* via a green synthesis approach. The synthesized CuNPs were characterized using UV-visible spectroscopy, Fourier-transform infrared spectroscopy (FTIR), gas chromatography–mass spectrometry (GC-MS), field-emission scanning electron microscopy coupled with energy-dispersive X-ray analysis (FESEM-EDAX), X-ray diffraction (XRD), and thin-layer chromatography (TLC). Phytochemical screening was performed to identify the biomolecules involved in reduction and capping. Biological potential of the polyherbal CuNPs was further evaluated using antibacterial activity assays, 2,2-diphenyl-1-picrylhydrazyl (DPPH) antioxidant assay, cytotoxicity assessment using the MTT assay in HepG2 cells, and DNA fragmentation analysis, thereby providing a comprehensive evaluation of their potential biomedical relevance.

## 2. Materials and Methods

### 2.1. Biosynthesis of CuNPs

All chemicals used in this study were of analytical grade. Fresh plant materials of *H. auriculata* and *L. aspera* were collected from different locations in Chennai, Tamil Nadu, India. The plant samples were thoroughly washed with distilled water, shade-dried at room temperature, and ground into a fine powder. The powdered materials were stored separately in airtight containers at room temperature until further use.

For preparation of the polyherbal extract, 5 g of each dried plant powder was added separately to 50 mL of distilled water and heated at 70–80 °C for 20 min using a heating mantle (Krishnaram Scientific, Chennai, India). The extracts were cooled to room temperature and filtered through Whatman No. 1 filter paper. Equal volumes (25 mL each) of *H. auriculata* and *L. aspera* extracts were mixed in a 1:1 ratio to obtain the polyherbal extract.

**Figure 1:**
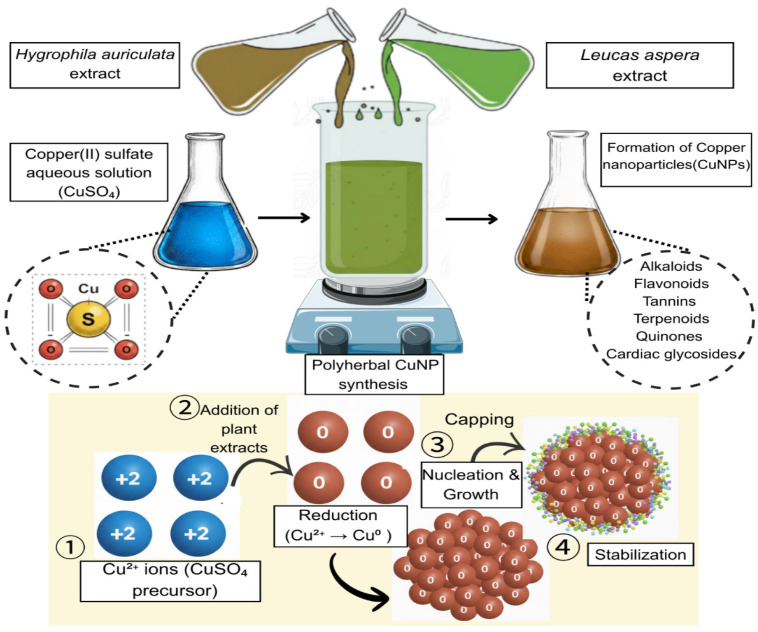
Polyherbal green synthesis of copper-based nanoparticles (CuNPs) using *Hygrophila auriculata* and *Leucas aspera*.

Separately, a 0.5 M aqueous solution of copper sulfate (50 mL) was prepared and added dropwise to the polyherbal extract in a 1:1 ratio under continuous stirring using a magnetic stirrer (Krishnaram Scientific, Chennai, India) (Figure 1). The reaction mixture was incubated at 37 °C for 24 h. CuNP formation was indicated by a visible color change in the reaction mixture. The synthesized nanoparticles were subsequently lyophilized for further characterization and evaluation of their application potential [26,27,28,29].

### 2.2. Phytochemical Screening of Plant Extracts

Preliminary phytochemical screening of each plant extract was performed separately using standard qualitative assays for alkaloids, saponins, and flavonoids (Shinoda test), tannins and terpenoids (Salkowski test), and quinones and cardiac glycosides (Keller–Killiani test) according to previously reported protocols [30,31,32].

### 2.3. Characterization of Synthesized CuNPs

Polyherbal green-synthesized CuNPs were initially analyzed using UV-visible spectroscopy with a double-beam spectrophotometer (Systronics, Ahmedabad, India). Absorbance spectra were recorded in the wavelength range of 200–800 nm to monitor nanoparticle formation [33].

Surface morphology and particle size of the synthesized CuNPs were examined using FESEM. FESEM Images were obtained using an Apreo 2 S HiVac FESEM system (Thermo Fisher Scientific, Waltham, MA, USA) operated at an accelerating voltage of 15 kV. Signals were detected using an Everhart–Thornley detector (TESCAN SEA Pte. Ltd, The Gateway East Tower, Singapore) at a magnification of 100,000×. Elemental composition of the nanoparticles was determined by energy-dispersive X-ray spectroscopy (EDS) using an Oxford Instruments EDS system (Oxford Instruments, Bristol, UK) coupled to the FESEM instrument [34].

FTIR was used to identify functional groups associated with the surface of the synthesized CuNPs. FTIR spectra were recorded using a Bruker Fourier-transform infrared spectrometer (Bruker Optics, Ettlingen, Germany) equipped with an attenuated total reflectance accessory employing a ZnSe crystal and an RT-DLATGS detector. Spectra were collected at a resolution of 2 cm^−1^ in the range of 400–4000 cm^−1^ [35].

GC-MS analysis was conducted to identify phytochemical constituents present in the polyherbal extract using an Agilent 7890A gas chromatograph coupled with a mass spectrometer (Agilent Technologies, Santa Clara, CA, USA). The analysis was performed in full-scan mode over a mass range of 1–455 *m*/*z*, with a total run time of 47.95 min [36,37].

XRD analysis was performed to determine crystalline structure, phase composition, and average crystallite size of the synthesized CuNPs. Diffraction patterns were recorded using a PANalytical Empyrean X-ray diffractometer (Malvern PANalytical, Almelo, The Netherlands) equipped with a PIXcel3D detector and Cu Kα radiation (λ = 1.5406 Å) over a 2θ range of 10–80°. Average crystallite size was estimated using the Scherrer equation [34].

### 2.4. Antibacterial Activity of Plant Extracts and CuNPs

Antibacterial activity of the synthesized CuNPs was evaluated using the standard disc diffusion method. Four pathogenic bacterial strains were used: *Escherichia coli* (ATCC 25922), *Pseudomonas aeruginosa* (ATCC 27853), *Staphylococcus aureus* (ATCC 25923), and *Streptococcus pyogenes* (ATCC 19615). The bacterial strains were obtained from the Department of Microbiology, Vellore Institute of Technology (VIT), Vellore, India.

Bacterial suspension (100 µL) was uniformly spread onto Mueller–Hinton agar plates. Three wells (approximately 6 mm in diameter) were punched into the agar and, respectively, filled with 100 µL of sterilized *L. aspera* extract, *H. auriculata* extract (100 mg/mL each), and copper nanoparticles (100 µg/mL). Prior to use, plant extracts and CuNP suspensions were filtered through Whatman No. 1 filter paper to remove debris and sterilized using a 0.22 µm membrane filter (Membrane Hitec: Chennai, India).

Positive and negative controls were included using discs impregnated with ceftazidime (30 µg) and distilled water, respectively. The plates were incubated at 37 °C for 24 h; diameters of the zones of inhibition were measured in millimeters. All experiments were performed in triplicate [26].

### 2.5. In Vitro Antioxidant Assay of CuNPs

**Figure 2:**
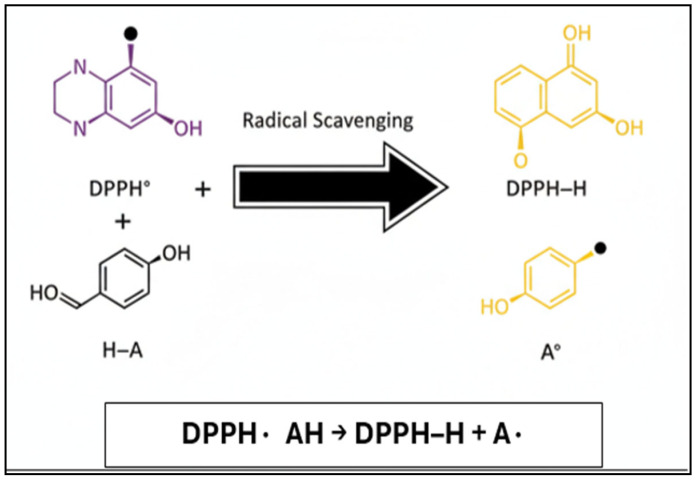
Schematic representation of the 2,2-diphenyl-1-picrylhydrazyl (DPPH) radical scavenging mechanism, illustrating reduction in the DPPH radical (DPPH•) to its non-radical form (DPPH–H) via hydrogen atom or electron donation by antioxidant molecules.

Activity was evaluated using the DPPH free radical scavenging assay. DPPH is a stable nitrogen-centered free radical commonly used to assess antioxidant capacity. The reduction in DPPH• to its scavenged form (DPPH-H) occurs via hydrogen atom donation from antioxidant compounds (Figure 2).

A 0.1 mM DPPH solution was prepared by dissolving 4 mg of DPPH in 100 mL of ethanol. Different concentrations of the sample extracts (20–200 µg/mL) were prepared using dimethyl sulfoxide (DMSO) and adjusted to a final volume of 40 µL. Subsequently, 2.96 mL of the DPPH solution was added; the reaction mixture was incubated in the dark at room temperature for 20 min. After incubation, absorbance was measured at 517 nm using a UV-visible spectrophotometer (Hitachi, Tokyo, Japan). The control consisted of 3 mL of DPPH solution without the sample.

All antioxidant experiments were performed in triplicate (*n* = 3). The results are expressed as mean ± standard deviation (SD). Statistical analysis was conducted using one-way analysis of variance (ANOVA) followed by Tukey’s post hoc test to determine significant differences between treated samples and the control.

Radical scavenging activity (%) was calculated using the following equation [38,39]:

$$\mathrm{Radical}\;\mathrm{scavenging}\;\mathrm{activity}\;(\%)\;=\;\;\frac{Absorbance\;control\;-\;Absorbance\;sample}{Absorbance\;control}\;\;\times 100$$

### 2.6. Cytotoxicity Assay of CuNPs

Cytotoxicity of the polyherbal green-synthesized CuNPs was evaluated using the HepG2 human liver cancer cell line obtained from the National Centre for Cell Sciences (NCCS), Pune, India. The MTT assay was used to determine cell viability.

HepG2 cells were cultured in Dulbecco’s Modified Eagle’s Medium (DMEM) supplemented with 10% fetal bovine serum and maintained at 37 °C in a humidified incubator containing 5% carbon dioxide (CO_2_). Cells were harvested using trypsin-EDTA and seeded into 96-well plates at a density of approximately 1 × 10^4^ cells per well. After incubation for 24 h to allow cell attachment, the medium was replaced with serum-free DMEM containing CuNPs at different concentrations; untreated cells served as the control.

Following 24 h of treatment, 100 µL of MTT solution (0.5 mg/mL) was added to each well and incubated for 4 h at 37 °C. The resulting formazan crystals were dissolved in 100 µL of DMSO; absorbance was measured at 570 nm using a microplate reader (EPS Biosolutions, Chennai, India), with 630 nm as the reference wavelength. All experiments were performed in triplicate (*n* = 3). The results are expressed as mean values [40,41].

Cell viability (%) was calculated using the following formula:

$$\mathrm{Cell}\;\mathrm{viability}\;(\%)\;=\;\frac{Sample\;OD}{Control\;OD}\;\;\times 100$$

### 2.7. Analysis of DNA Fragmentation

DNA fragmentation analysis was performed to evaluate apoptosis in HepG2 cells following treatment with polyherbal green-synthesized CuNPs. The assay was conducted according to previously reported methods, with minor modifications.

HepG2 cells were treated with CuNPs at half maximal inhibitory concentration (IC_50_) determined by the MTT assay and incubated for 24 h at 37 °C under humidified conditions containing 5% CO_2_ in serum-free medium. After treatment, cells were harvested by trypsinization, washed with phosphate-buffered saline, and subjected to genomic DNA isolation using a TrueScreen DNA Isolation Kit (Aura Biotechnologies Private Limited, Chennai, India) according to the manufacturer’s instructions.

The cell pellet was resuspended in lysis buffer, added lysozyme and proteinase K, and incubated at 65 °C to ensure complete cell lysis and protein digestion. Genomic DNA was purified using a spin column–based method involving sequential washing steps and subsequently eluted in elution buffer.

The isolated DNA samples were analyzed by agarose gel electrophoresis using a 1% agarose gel prepared in 1 × TAE buffer containing ethidium bromide. A DNA ladder was loaded into the first well as a molecular size marker. Subsequent wells contained DNA from HepG2 cells treated with concentrations below the IC_50_, at the IC_50_, and untreated cells (negative control). Electrophoresis was performed under appropriate voltage conditions; DNA bands were visualized using a UV transilluminator (EPS Biosolutions, Chennai, India) [42].

Densitometric analysis of agarose gel images was performed using ImageJ (version 1.8.0) to quantify DNA fragmentation. Pixel densities corresponding to intact genomic DNA bands and fragmented DNA smears were measured after background subtraction; the percentage of fragmentation was calculated using the following formula [43]:

$$\mathrm{Fragmentation}\;\%\;=\;\frac{Pixel\;density\;of\;Fragmented\;Smear}{Pixel\;density\;of\;intact\;band+Pixel\;density\;of\;Fragmented\;Smear}\;\times 100$$

### 2.8. TLC

TLC was performed to compare phytochemical profiles of the polyherbal extract and synthesized CuNPs. The plant extract and nanoparticle suspension were applied onto aluminum-backed TLC plates.

The mobile phase consisted of chloroform and methanol in a 1:2 ratio. After chromatographic development, the separated compounds were visualized using iodine vapor. The plates were subsequently examined under UV illumination to observe the migration of phytochemical constituents [44,45].

### 2.9. Statistical Analysis

All results are presented as mean ± SD. Statistical analysis was performed using one-way ANOVA followed by Tukey’s post hoc test. Differences were considered statistically significant at *p* < 0.05.

## 3. Results and Discussion

### 3.1. Phytochemical Screening

**Figure 3:**
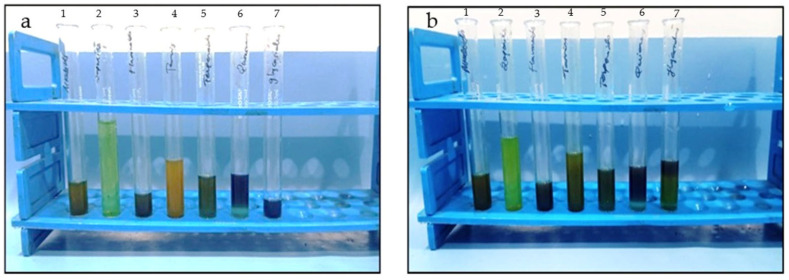
Qualitative phytochemical profiles of: (**a**) *L. aspera* and (**b**) *H. auriculata* leaf extracts. The figure represents qualitative detection of major secondary metabolites, including alkaloids, flavonoids, tannins, terpenoids, quinones, and cardiac glycosides, based on standard phytochemical screening tests.

Phytochemical screening of *L. aspera* and *H. auriculata* leaf extracts revealed the presence of alkaloids, flavonoids, tannins, terpenoids, quinones, and cardiac glycosides; saponins were absent in both extracts (Table 1; Figure 3a,b). Strong positive reactions (+++) were observed for flavonoids, tannins, alkaloids, and quinones; terpenoids and cardiac glycosides exhibited moderate presence (++). Qualitative phytochemical profiles of the two plant extracts were largely comparable, with only minor variations in relative intensity of the detected compounds.

The phytochemical composition observed in *L. aspera* and *H. auriculata* is consistent with the findings of previous reports describing their rich secondary metabolite profiles [46,47]. The strong presence of flavonoids suggests their potential involvement as reducing and capping agents during the green synthesis of CuNPs. Flavonoids donate electrons and stabilize nanoparticle surfaces via hydroxyl and carbonyl functional groups.

Additionally, the detection of tannins and quinones supports antioxidant and antimicrobial potential of the plant extracts. These compounds are known to contribute to redox activity and microbial inhibition, which may enhance biological performance of the synthesized nanoparticles. The presence of cardiac glycosides and terpenoids further highlights medicinal relevance of the selected plants. Overall, the phytochemical richness of *L. aspera* and *H. auriculata* supports their suitability for polyherbal-mediated nanoparticle synthesis, with potential biological functionality.

### 3.2. UV-Visible Spectroscopy

**Figure 4:**
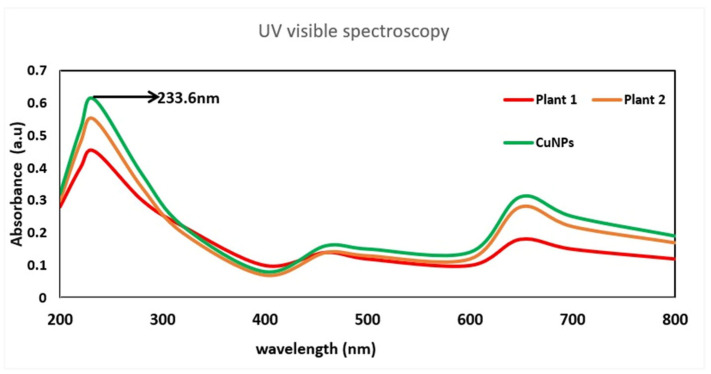
UV-visible absorption spectra of *H. auriculata* leaf extract (plant 1), *L. aspera* leaf extract (plant 2), and polyherbal-mediated CuNPs recorded in the range of 200–800 nm. The CuNPs exhibited a characteristic absorption peak at 233.6 nm, confirming nanoparticle formation, whereas the plant extracts exhibited UV-region absorption attributed to phytochemical constituents.

UV-visible spectroscopy performed in the range of 200–800 nm revealed a characteristic absorption peak at 233.6 nm (Figure 4), indicating CuNP formation. The distilled water control exhibited no significant absorption within the measured range. CuNPs synthesized using the polyherbal extract exhibited higher absorbance than those synthesized using the individual plant extracts, suggesting effective nanoparticle formation and stabilization by bioactive phytochemicals.

The absorption peak at 233.6 nm may be attributed to electronic transitions associated with copper-oxygen species such as Cu_2_O or CuO, as well as contributions from phytochemicals present in the plant extract, rather than with the classical surface plasmon resonance (SPR) of metallic CuNPs, which typically occurs at approximately 550–600 nm. Absence of a distinct SPR peak in the visible region may be associated with small particle size, partial oxidation, or surface capping by organic molecules. Compared with the individual plant extracts, the synthesized nanoparticles exhibited higher absorbance intensity, further supporting the formation of CuNPs stabilized by polyherbal phytochemicals. Similar observations have been reported in previous studies on the plant-mediated synthesis of CuNPs, where phytochemicals are found to play a critical role in reduction and stabilization [26,48,49,50].

### 3.3. FTIR

**Figure 5:**
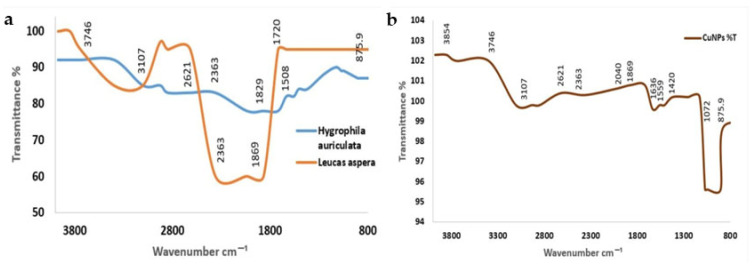
Fourier-transform infrared spectroscopy (FTIR) spectra showing peak positions and corresponding functional groups involved in the polyherbal-mediated synthesis of CuNPs. (**a**) FTIR spectra of the polyherbal *H. auriculata* and *L. aspera* extracts, indicating major functional groups responsible for reduction and stabilization. (**b**) FTIR spectrum of synthesized CuNPs, confirming the involvement of phytochemicals in nanoparticle formation and capping.

FTIR spectral analysis was performed to identify functional groups associated with the polyherbal-mediated CuNPs based on their characteristic absorption bands (Figure 5). The observed peaks and their corresponding functional groups are summarized in Table 2. The FTIR spectrum displayed bands corresponding to hydroxyl (–OH), carbonyl (C=O), amine (N–H), alkene (C=C), and C–O functional groups. These findings suggest the presence of phenolic and alcoholic compounds that may participate in nanoparticle reduction and stabilization.

The detection of carbonyl- and amine-related bands may indicate the involvement of phytoconstituents such as flavonoids and other bioactive compounds in capping [51,52]. Overall, the FTIR results indicate presence of multiple functional groups within the polyherbal extract that can interact with the nanoparticle surface. These functional moieties may contribute to the reduction of copper ions and stabilization of nanoparticles by surface binding.

### 3.4. GC-MS Analysis

**Figure 6:**
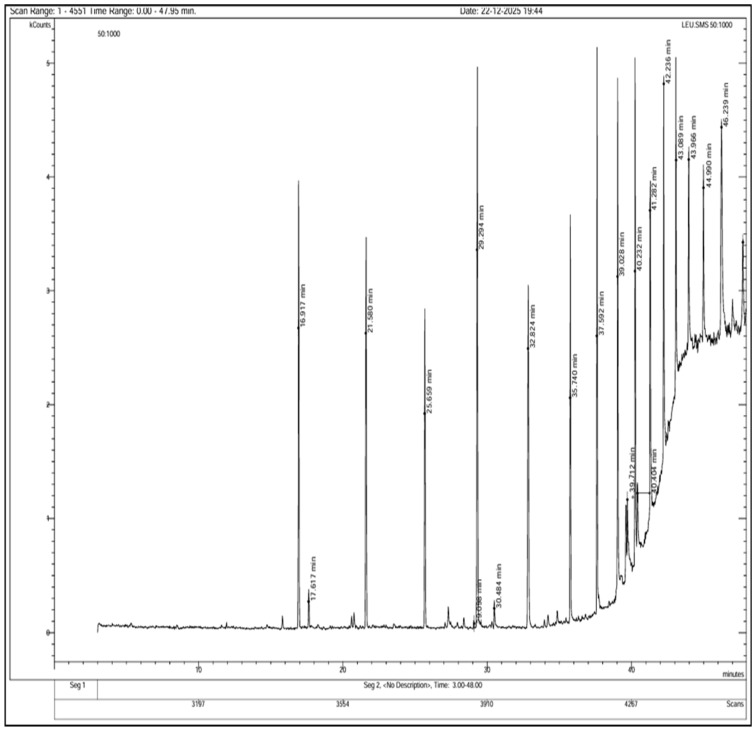
Gas chromatography–mass spectrometry (GC-MS) chromatogram of the polyherbal leaf extract showing the distribution of phytochemical constituents. Multiple peaks were observed between retention times of 16.9 and 47.9 min, corresponding to aromatic compounds, phenolics, organic acids, phosphonic acid derivatives, amide-containing compounds, and sulfur-containing compounds.

GC-MS analysis of the polyherbal extract revealed a complex phytochemical profile, with multiple peaks observed between retention times of 16.9 and 47.9 min in the chromatogram (Figure 6). Tentatively identified compounds included aromatic compounds, phenolic compounds, organic acids, phosphonic acid derivatives, amide-containing compounds, and sulfur-containing compounds (Table 3). Major constituents included benzene and naphthalene derivatives, acetic acid esters, ethylphosphonic acid derivatives, thiophenecarboxylic acid, propenamide derivatives, and phenolic compounds such as 2,4-dihydroxypropiophenone, indicating the presence of diverse bioactive secondary metabolites.

The diverse phytochemical constituents identified by GC-MS are likely to play a critical role in the green synthesis of CuNPs. Phenolic compounds and organic acids can act as reducing agents via electron donation and are well known for their antioxidant properties [53]. The presence of thiophene derivatives may contribute to antibacterial activity of the polyherbal-mediated CuNPs; such compounds disrupt bacterial cell membranes and interfere with metabolic pathways [54]. Additionally, naphthalene derivatives and steroidal oximes detected in the extract are associated with antimicrobial and anticancer activities, particularly those against HepG2 cells [55,56].

**Figure 7:**
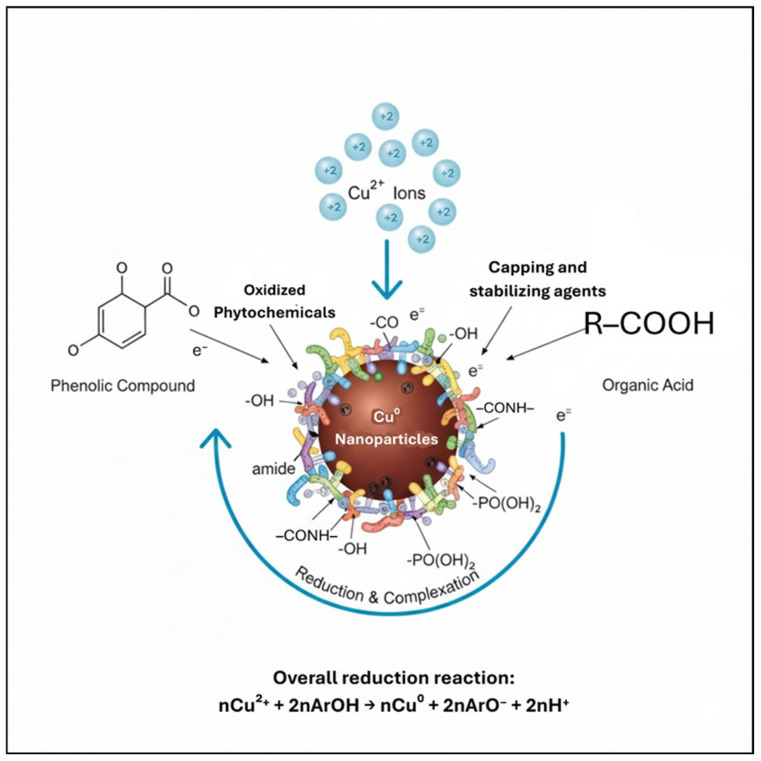
Schematic representation of the polyherbal-mediated green synthesis of copper nanoparticles (CuNPs), illustrating the role of phytochemicals identified by GC–MS in the reduction of Cu^2+^ ions and the subsequent capping and stabilization of CuNPs. Phenolic compounds, organic acids, and other functional groups (e.g., hydroxyl, carbonyl, amide, and phosphonic moieties) act as reducing, complexing, and stabilizing agents, consistent with the functional group analysis obtained from FTIR spectroscopy.

Functional groups such as hydroxyl, carbonyl, amide, and phosphonic moieties identified by GC-MS can interact with the nanoparticle surface, acting as capping and stabilizing agents (Figure 7) [57,58,59]. These findings complemented the FTIR results, which showed characteristic absorption bands corresponding to O–H, C=O, N–H, and C–O functional groups. Collectively, GC-MS and FTIR analyses confirm the involvement of plant-derived biomolecules in the reduction, capping, and stabilization of CuNPs. Diverse phytochemical composition of the polyherbal extract may contribute to nanoparticle stability and biological activity.

### 3.5. FESEM-EDAX

**Figure 8:**
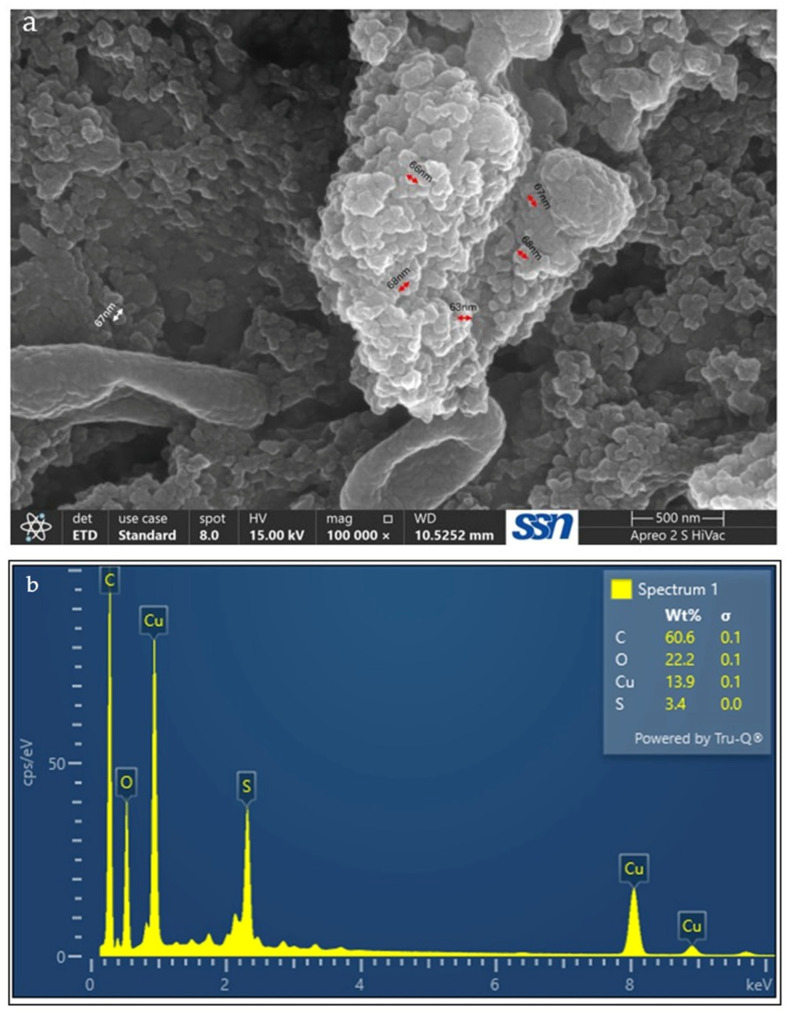
Field-emission scanning electron microscopy (FESEM) and energy-dispersive X-ray (EDAX) analyses of polyherbal-synthesized CuNPs. (**a**) FESEM micrograph showing predominantly spherical to quasi-spherical morphology with good dispersion and an average particle size of 66.5 ± 1.87 nm. (**b**) EDAX spectrum confirming copper as a major elemental component, along with oxygen, carbon, and sulfur, indicating the presence of phytochemical residues on the nanoparticle surface.

FESEM analysis was performed to examine morphology and particle size of the polyherbal-synthesized CuNPs (Figure 8a). FESEM images revealed that the nanoparticles exhibited predominantly spherical to quasi-spherical morphology with relatively good dispersion. Average particle size was calculated as 66.5 ± 1.87 nm using ImageJ. The observed surface morphology indicates the formation of crystalline nanoscale particles.

EDAX analysis (Figure 8b) confirmed the presence of copper as a major elemental component, along with oxygen, carbon, and sulfur. The detection of these elements suggests the formation of copper nanoparticles with phytochemical residues associated with the nanoparticle surface.

The spherical to quasi-spherical morphology observed in the FESEM images is consistent with the findings of previous reports on plant-mediated green synthesis of CuNPs, where phytochemicals are found to act as reducing and capping agents that influence particle growth [60,61]. The relatively uniform morphology observed in the present study may be associated with the presence of multiple phytoconstituents in the polyherbal extract, which may interact with the nanoparticle surface during nucleation and growth.

Compared with monoherbal systems, the presence of diverse biomolecules in polyherbal extracts may enhance surface passivation, potentially contributing to improved morphological homogeneity. The average particle size obtained in this study falls within the nanoscale range reported for CuNPs synthesized using leaf extracts of *Hyptis suaveolens*, *Fortunella margarita*, and *Eucalyptus globulus*, which typically range from 50 to 75 nm [62,63,64]. The relatively narrow size distribution suggests controlled nanoparticle growth, possibly due to cooperative capping effects from multiple phytochemical classes that limit aggregation.

EDAX results further support the green synthesis mechanism. The presence of carbon and oxygen alongside copper is commonly observed in plant-mediated CuNP synthesis and may be attributed to phytochemicals acting as capping and stabilizing agents [33]. The detection of sulfur may indicate the involvement of sulfur-containing heterocyclic compounds that can contribute to nanoparticle stability and biological activity.

### 3.6. XRD Analysis

**Figure 9:**
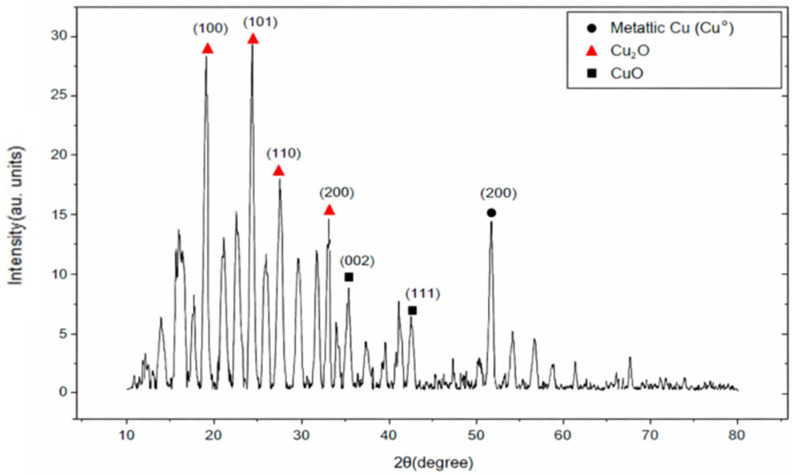
X-ray diffraction (XRD) pattern of the synthesized CuNPs. The indexed diffraction peaks correspond to Cu_2_O (▲), metallic Cu (●), and CuO (■) phases. Peak positions were matched with standard JCPDS cards: Cu (04-0836), Cu_2_O (05-0667), and CuO (48-1548), confirming mixed-phase composition of the sample.

XRD analysis was performed to examine crystalline structure and phase composition of the polyherbal-synthesized CuNPs. The corresponding diffraction pattern is shown in Figure 9. The XRD pattern confirmed crystalline nature of the synthesized material and revealed the presence of multiple copper-based phases.

Crystallite size was calculated using the Debye–Scherrer equation:

D = Kλ/(β cos θ)

where K = 0.9, λ = 0.15406 nm (Cu Kα radiation), β represents the full width at half maximum (FWHM), and θ denotes the Bragg angle (Table 4). Crystallite sizes calculated from the major diffraction peaks were 11.4, 11.4, 10.7, 13.5, 10.0, 12.1, and 10.1 nm, resulting in an average crystallite size of approximately 11.5 nm.

XRD analysis confirmed the presence of metallic copper, along with Cu_2_O and a minor CuO phase. Although the reduction in Cu^2+^ ions to Cu^0^ was substantial, it was not complete. The Cu_2_O phase may result from the partial reduction in Cu^2+^ to Cu^+^, whereas CuO and Cu_2_O phases may also form owing to the surface oxidation of CuNP during drying or storage.

The diffraction peaks were indexed using standard JCPDS reference cards: Cu (04-0836), Cu_2_O (05-0667), and CuO (48-1548), confirming mixed-phase nature of the synthesized nanoparticles. Most prominent peaks corresponded to Cu_2_O and Cu, whereas CuO was present as a minor phase. Weak unindexed reflections were likely associated with nanoscale effects, minor surface oxidation, or instrumental background; no additional crystalline impurity phases were detected within the instrumental detection limits.

Multiphase crystalline structure observed in the XRD pattern is consistent with the findings of previous reports on green-synthesized CuNPs, where metallic copper and oxide phases are found to coexist [65,66,67]. Average crystallite size in the nanoscale range suggests controlled crystal growth under the given synthesis conditions. The relatively small crystallite size may be influenced by phytochemicals present in the extract, which may act as stabilizing agents and limit excessive crystal growth. However, additional comparative studies are required to determine specific influence of the polyherbal system on structural regulation [68,69].

### 3.7. TLC

**Figure 10:**
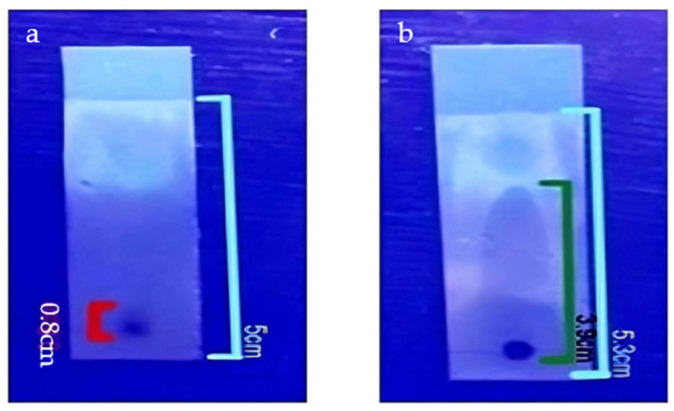
Thin-layer chromatography (TLC) analysis showing the retention factor (R_f_) values of (**a**) polyherbal-synthesized CuNPs and (**b**) polyherbal plant extract. R_f_ values were 0.16 for the CuNPs and 0.74 for the plant extract; R_f_ values were calculated based on distances traveled by the solvent front and respective substances.

TLC was performed to compare the mobility of phytochemicals present in the plant extract and synthesized CuNPs (Figure 10). Both the plant extract and CuNP suspension were prepared in distilled water; 5 µL of each sample was spotted onto aluminum-backed silica gel TLC plates. The plates were developed using an appropriate solvent system; retention factor (R_f_) was calculated using the following equation [70]:

$$R_{f}=\frac{\mathrm{Distance}\;\mathrm{travelled}\;\mathrm{by}\;\mathrm{the}\;\mathrm{substance}}{\mathrm{Distance}\;\mathrm{travelled}\;\mathrm{by}\;\mathrm{the}\;\mathrm{solvent}\;\mathrm{front}}$$

The calculated R_f_ values reflected relative mobility of the analyzed components. The plant extract exhibited an elevated R_f_ value (0.74), whereas the nanoparticle suspension exhibited minimal migration, with a R_f_ value of 0.16. Limited mobility of the nanoparticle sample may be attributed to enhanced interactions with the stationary phase and reduced diffusion owing to the large size and high polarity of nanoparticle-associated phytochemicals.

Similar trends have been reported in previous TLC analyses of plant-mediated nanoparticle systems, where free phytochemical constituents in plant extracts are found to display higher mobility than nanoparticle-associated compounds [71]. These observations suggest that phytochemicals conjugated to the CuNPs may enhance adsorption to the stationary phase and reduce chromatographic mobility.

### 3.8. Antibacterial Activity

**Figure 11:**
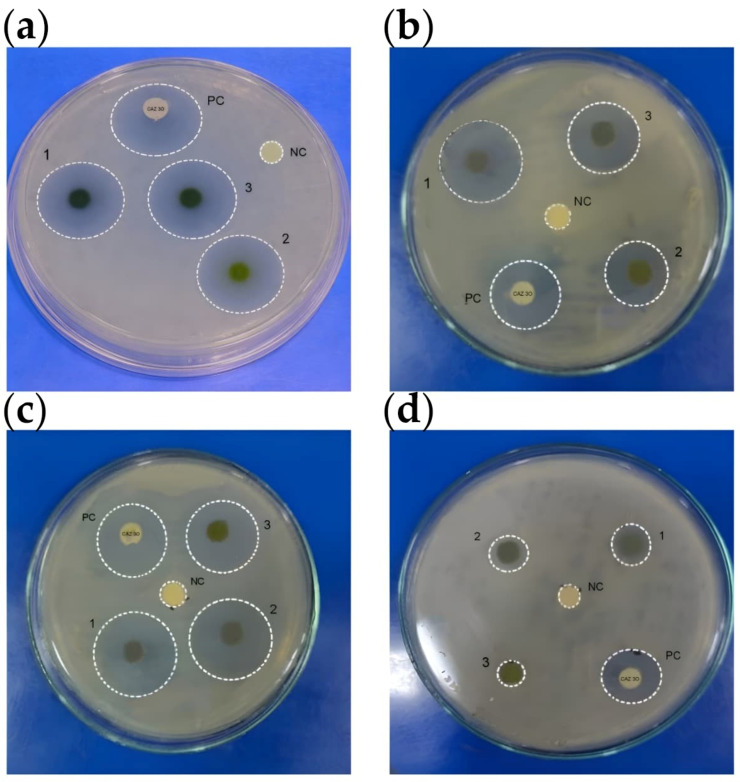
Antibacterial activity of polyherbal-synthesized copper nanoparticles (CuNPs) against selected pathogenic bacteria, evaluated using the agar well diffusion method and expressed as zone of inhibition (mm). The experiments were carried out using plant extracts at 100 mg/mL and CuNPs at 100 µg/mL. (**a**) *Staphylococcus aureus*, (**b**) *Pseudomonas aeruginosa*, (**c**) *Escherichia coli*, and (**d**) *Streptococcus pyogenes*. The treatments of CuNPs and plant extracts were represented in numbers, where No. 1 corresponds to polyherbal-CuNPs, No. 2 to *Leucas aspera* extract and No. 3 to *Hygrophila auriculata* extract. NC represents the negative control (sterile water), while PC represents the positive control (CAZ-30 - ceftazidime, 30 µg/mL). The zones of inhibition observed around each well indicate the antibacterial effect against the respective bacterial strains, with CuNPs showing a more pronounced activity compared to the individual plant extracts.

Antibacterial activity of the synthesized samples was evaluated using the zone of inhibition method (Figure 11). The corresponding inhibition diameters are summarized in Table 5. Among the tested samples, the polyherbal-mediated CuNPs exhibited the highest antibacterial activity against all tested bacterial strains. The largest zones of inhibition were observed against *S. aureus* (20 mm) and *E. coli* (28 mm), indicating strong antibacterial activity.

Enhanced antibacterial activity of the synthesized CuNPs is consistent with the findings of previous reports demonstrating the effectiveness of CuNPs against Gram-positive and Gram-negative bacteria [72,73]. Considering that the synthesized material exhibits a multiphase composition comprising Cu_2_O, CuO, and metallic Cu, the observed antibacterial activity may arise from the combined contributions of these phases rather than from metallic copper alone.

Compared with monoherbal green synthesis systems, the polyherbal approach may provide a broader spectrum of bioactive phytochemicals. Antibacterial activity of the plant extracts themselves may be attributed to phytochemical constituents such as phenolics, flavonoids, alkaloids, and terpenoids, which disrupt bacterial cell membranes and interfere with essential cellular processes [46,47,74].

**Figure 12:**
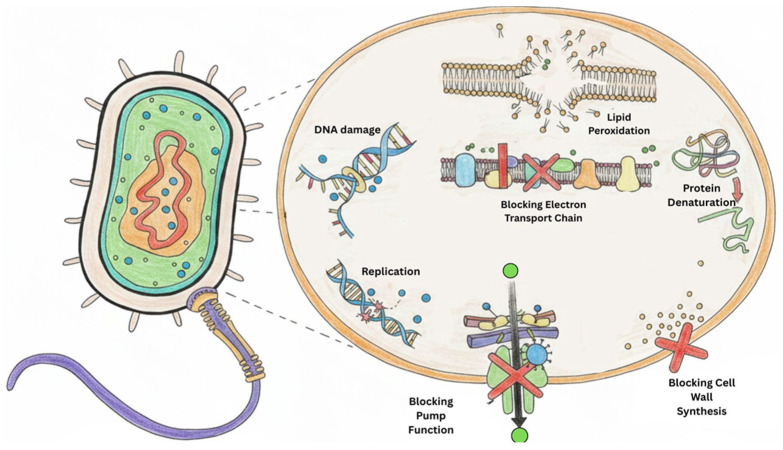
Proposed antibacterial mechanism of action of polyherbal-synthesized CuNPs. CuNPs interact with bacterial cell surfaces, disrupt cell wall and membrane integrity, generate reactive oxygen species, and interfere with essential cellular processes, including DNA replication, electron transport, protein function, and lipid metabolism, ultimately leading to bacterial cell death.

The antibacterial efficacy of CuNPs may also be associated with their nanoscale dimensions, which facilitate close interaction with bacterial cells. CuNPs adhere to bacterial cell walls, disrupt membrane integrity, and generate reactive oxygen species, leading to oxidative stress, lipid peroxidation, protein denaturation, and ultimately, bacterial cell death (Figure 12) [75]. However, the precise antibacterial mechanism in the present study requires further investigation. Surface-bound phytochemicals originating from the polyherbal extract may additionally influence nanoparticle-cell interactions and contribute to the observed antibacterial activity.

### 3.9. In Vitro Antioxidant Assay

Antioxidant activity of the polyherbal CuNPs was evaluated using the DPPH radical scavenging assay at concentrations of 10, 20, 30, 40, and 50 mg/mL. The nanoparticles exhibited a concentration-dependent increase in DPPH radical scavenging activity (Table 6). Correspondingly, optical density (OD) values decreased progressively with increasing nanoparticle concentration (Table 7), indicating enhanced scavenging of DPPH radicals.

Statistical analysis revealed that the absorbance observed at higher concentrations (30–50 mg/mL) was significantly reduced (*p* < 0.05) compared with that in the control. The increased antioxidant activity at elevated nanoparticle concentrations may be attributed to surface-bound phytochemicals, particularly phenolic and flavonoid compounds, which participate in hydrogen atom donation and electron transfer reactions. Similar concentration-dependent DPPH radical scavenging activity has been reported for other green-synthesized CuNPs, including those prepared using *Passiflora* flower extract [76]. Additionally, CuNPs synthesized using *Moringa oleifera* leaf extracts have demonstrated comparable concentration-dependent antioxidant activity [45]. These findings suggest that the synthesized nanoparticles possess measurable antioxidant potential.

### 3.10. Cytotoxicity Assay

Cytotoxic potential of the polyherbal CuNPs was evaluated against HepG2 cells using the MTT assay. A concentration-dependent reduction in cell viability was observed after 48 h of treatment. Cell viability decreased from 89.2 ± 0.96% at 15.62 µg/mL to 32.95 ± 1.70% at 250 µg/mL compared with that of the untreated control cells (100%) (Table 8). Approximately 50% cell viability (IC_50_) was observed at a concentration of 125 µg/mL, indicating moderate cytotoxic activity against HepG2 cells (Figure 10).

**Figure 13:**
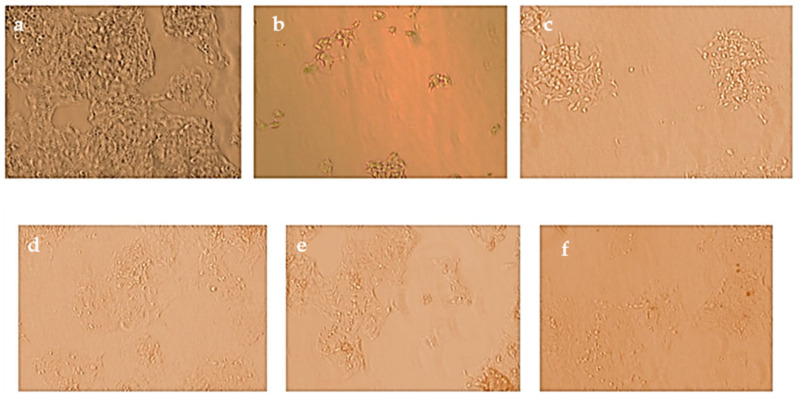
Morphological changes in HepG2 cells following treatment with polyherbal-synthesized CuNPs observed under a light microscope. (**a**) Untreated control cells exhibiting normal morphology and high confluency. (**b**–**f**) Cells treated with decreasing CuNP concentrations: (**b**) 250 µg/mL; (**c**) 125 µg/mL; (**d**) 62.5 µg/mL; (**e**) 31.25 µg/mL; and (**f**) 15.62 µg/mL. The size measurement of scale bar line is 500 µm (**a**–**f**).

**Figure 14:**
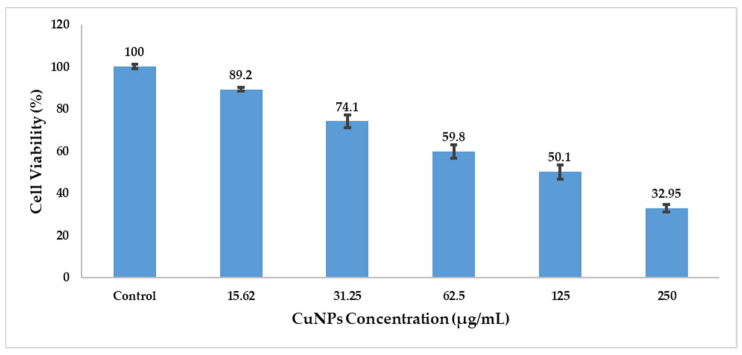
Effect of polyherbal-synthesized CuNPs on cell viability following 48 h exposure. Cells were incubated at 37 °C in a humidified atmosphere containing 5% carbon dioxide (CO_2_); cell viability (%) was determined at different CuNP concentrations using untreated cells as the control. The values are presented as mean ± SD of three independent experiments.

Statistical analysis revealed that CuNPs significantly reduced cell viability at concentrations ≥ 62.5 µg/mL (*p* < 0.05), whereas higher concentrations (125 and 250 µg/mL) produced highly significant cytotoxic effects (*p* < 0.01). The reduction in cell viability correlates with decreased mitochondrial metabolic activity, as indicated by reduced formazan formation (Figure 13 and Figure 14).

The dose-dependent cytotoxicity observed in HepG2 cells suggests that the polyherbal CuNPs possess potential anticancer activity. The observed reduction in cell viability may be associated with mitochondrial dysfunction and oxidative stress, which are key mechanisms underlying CuNP-induced cytotoxicity [77,78]. Compared with monoherbal nanoparticle systems, polyherbal-mediated CuNPs may exhibit altered biological activity owing to the presence of multiple phytochemicals that interact with copper ions during nanoparticle formation. These interactions may influence nanoparticle surface chemistry and contribute to the observed cytotoxic effects.

### 3.11. DNA Fragmentation Analysis

**Figure 15:**
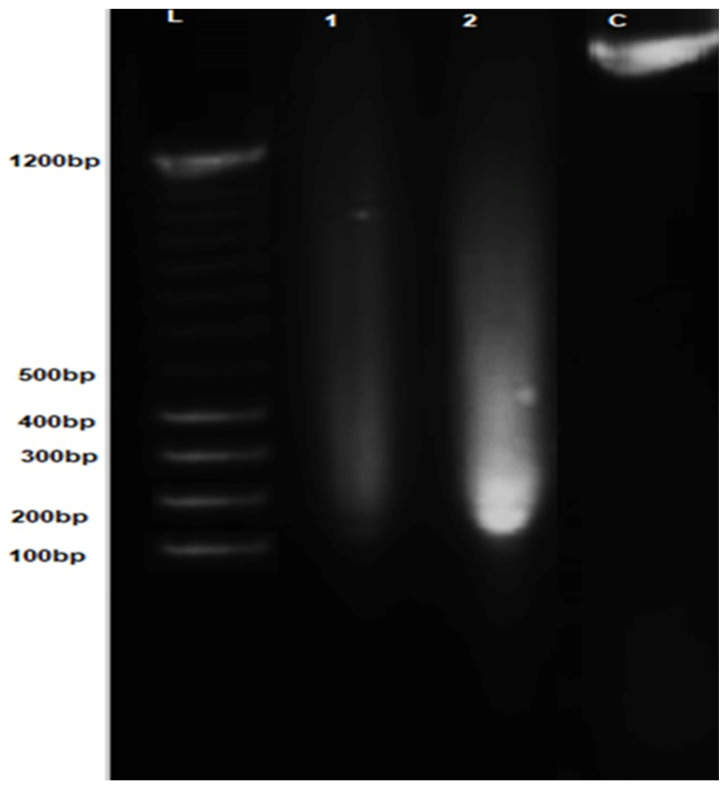
DNA fragmentation analysis of HepG2 cells treated with polyherbal-synthesized CuNPs. Lane L: DNA molecular weight marker; lane 1: HepG2 cells treated with a reduced concentration of CuNPs; lane 2: HepG2 cells treated at the IC_50_ concentration; lane C: negative control (crude DNA).

DNA fragmentation analysis was performed to investigate the mode of cell death induced by polyherbal CuNPs in HepG2 cells. Treated cells exhibited a pronounced fragmented DNA smear rather than a single intact genomic DNA band, indicating substantial DNA damage compared with untreated cells (Figure 15).

Densitometric analysis of the agarose gel images using ImageJ revealed that approximately 90–95% of the total DNA was present in fragmented form in cells treated at the IC_50_, as calculated using the fragmentation percentage formula [42,43].

DNA fragmentation is widely recognized as a biochemical hallmark of apoptosis, reflecting the endonuclease-mediated cleavage of chromosomal DNA [79]. The high degree of DNA fragmentation observed in IC_50_-treated HepG2 cells suggests that polyherbal CuNPs induce apoptotic DNA damage. These results are consistent with the dose-dependent reduction in cell viability observed in the MTT assay and indicate that cytotoxic effects of the nanoparticles may be primarily associated with apoptosis rather than with necrotic cell death.

## 4. Conclusions

An eco-friendly and sustainable polyherbal green synthesis of CuNPs was successfully achieved using *H. auriculata* and *L. aspera*. Preliminary phytochemical screening confirmed the presence of bioactive constituents in the individual plant extracts. Subsequent characterization using UV–Vis spectroscopy, FTIR, XRD, FESEM-EDAX, and GC-MS confirmed that the formation of CuNPs was reduced and stabilized by phytoconstituents present in the polyherbal extract. The nanoparticles exhibited predominantly spherical morphology, with an average crystallite size of approximately 11.5 nm. TLC revealed distinct R_f_ values for the plant extract (0.74) and CuNPs (0.16), indicating altered interactions with the stationary phase following nanoparticle formation.

The synthesized CuNPs exhibited antibacterial activity against *E. coli*, *P. aeruginosa*, *S. pyogenes*, and *Staphylococcus* spp. compared with the plant extract alone. FTIR and GC-MS analyses indicated the presence of bioactive phytoconstituents, including phenolics and esters, which may contribute to the observed antibacterial effects. Additionally, the CuNPs demonstrated concentration-dependent antioxidant activity in the DPPH assay and induced dose-dependent cytotoxic effects in HepG2 cells, as evaluated by the MTT assay.

Although in vitro cytotoxic effects were observed, the molecular mechanisms underlying nanoparticle interactions with cancer-related targets remain unresolved. Future studies integrating computational cancer biology approaches, such as molecular docking, molecular dynamics simulations, and in silico pathway analysis, may provide further insight into nanoparticle–protein interactions and potential structure–activity relationships. Such investigations, combined with experimental validation, will strengthen mechanistic understanding.

Overall, the findings suggest that polyherbal green-synthesized CuNPs are promising multifunctional nanomaterials with potential applications in sustainable nanomedicine and strategies aimed at addressing healthcare-associated infections.

## Figures and Tables

**Table 1:** Phytochemical screening results of *L. aspera* and *H. auriculata* leaf extracts.

Phytochemicals	*L. aspera*	*H. auriculata*
Alkaloids	+++	+++
Saponins	−	−
Flavonoids	+++	+++
Tannins	+++	+++
Terpenoids	++	++
Quinones	+++	+++
Cardiac Glycosides	++	++

− indicates compound absence; ++ indicates moderate presence; +++ indicates high presence.

**Table 2:** FTIR peak positions and corresponding functional groups of polyherbal-mediated CuNPs.

Wavenumber (cm^−1^)	Corresponding Functional Group	Vibration Type
3854	-OH	Stretching
3746	-OH	Stretching
3107	=C-H	Stretching
2621	S-H/-COOH	Stretching
2363	C≡C/CO_2_	Asymmetric stretching
2040	C≡C/C≡N	Stretching
1869	C=O	Stretching
1636	C=O	Amide I/flavonoids
1559	N-H	Bending (amide II)
1420	C-H/-COO-	Bending
1072	C-O	Stretching
875.9	Metal	Metal- oxygen vibration

**Table 3:** GC–MS–based tentative identification of phytochemical constituents present in the polyherbal leaf extract, showing retention time (RT), compound name, common name, peak area, relative abundance (%), and library match score (R.Match).

RT (min)	Compound Name	Common Name	Area	Amount (%)	R.Match
17.617	Benzene, 1-(1-methylethyl)-	Cumene (Isopropylbenzene)	1378	0.721	880
30.484	Acetic acid, 1,4-dihydroxy-9-octadecyl ester	Phenolic fatty acid ester	848	0.444	715
32.824	Ethylphosphonic acid, bis(trimethylsilyl) ester	Phosphonic acid derivative	14,030	7.345	826
39.600	2-Thiophenecarboxylic acid, 3-methyl	Thiophene derivative	1990	1.042	590
39.712	Propanamide, 2-(3-thienyl) amino	Amide-thiophene derivative	4102	2.147	560
46.239	1,4-Dimethyl-2-(2,6-diethyl) naphthalene	Alkyl-substituted naphthalene	13,204	6.912	555
47.011	16-Hydroxyimino-5-androsten-3-one	Steroidal oxime derivative	1473	0.771	598

**Table 4:** XRD peak positions (2θ), relative intensities, corresponding Miller indices (hkl), full width at half maximum (FWHM), and crystallite sizes of the synthesized-CuNPs.

2θ (°)	Intensity (a.u)	hkl	Crystallite Size D (nm)	FWHM
18.80	28.7273204	(100)/Cu_2_O	11.4	0.70113
24.10	29.407143	(101)/Cu_2_O	11.4	0.69649
27.15	17.2268219	(110)/Cu_2_O	10.7	0.74070
32.74	14.3441695	(200)/Cu_2_O	13.5	0.54619
35.19	8.59931039	(002)/CuO	10.0	0.79426
42.33	5.85144986	(111) Cu	12.1	0.53811
51.57	14.3505492	(200) Cu	10.1	0.73174

**Table 5:** Antibacterial activity of individual plant extracts (*L. aspera* and *H. auriculata*), the polyherbal extract, and the polyherbal-synthesized CuNPs against selected pathogenic bacteria at a concentration of 100 mg/mL (extracts) and 100 µg/mL (nanoparticles), expressed as zone of inhibition (mm). The values are presented as mean ± standard deviation (SD) (*n* = 3). Ceftazidime (30 µg/mL) was used as the positive control. ND indicates not determined. Statistical significance was considered at *p* < 0.05.

Organism	Positive Control (CAZ)	Negative Control	Plant 1	Plant 2	Polyherbal CuNPs
*S. pyogenes*	18 ± 1	0	14 ± 1	13 ± 1	20 ± 1
*E. coli*	30 ± 1	0	25 ± 1	26 ± 1	28 ± 1
*P. aeruginosa*	15 ± 1	0	10 ± 1	11 ± 1	13 ± 1
*S. aureus*	19 ± 1	0	18 ± 1	19 ± 1	20 ± 1

**Table 6:** DPPH radical scavenging activity (%) of polyherbal-synthesized CuNPs at different concentrations.

Concentration	Percentage of Activity
10 mg/mL	29.9%
20 mg/mL	41.8%
30 mg/mL	54.1%
40 mg/mL	60.5%
50 mg/mL	76.5%

**Table 7:** DPPH radical scavenging assay of polyherbal-synthesized CuNPs: optical density (OD) values at different concentrations, expressed as the mean ± SD of three independent trials.

Concentration	Trial-1 OD	Trial-2 OD	Trial-3 OD	Mean ± SD OD
10 mg/mL	0.698	0.697	0.698	0.698 ± 0.001
20 mg/mL	0.579	0.579	0.578	0.579 ± 0.001
30 mg/mL	0.457	0.457	0.457	0.457 ± 0.001
40 mg/mL	0.393	0.393	0.392	0.393 ± 0.001
50 mg/mL	0.234	0.233	0.234	0.234 ± 0.001
Control	0.996	0.997	0.996	0.996 ± 0.001

**Table 8:** Effect of polyherbal-synthesized CuNPs on the viability of HepG2 cells, expressed as mean OD ± SD and corresponding cell viability (%) at different concentrations.

Concentration (µg/mL)	Trial-1 (OD)	Trial-2 (OD)	Trial-3 (OD)	Mean OD ± SD	Cell Viability (%)
Control	0.091	0.093	0.095	0.093 ± 0.002	100.0 ± 1.08 ^a^
15.62	0.083	0.084	0.082	0.083 ± 0.001	89.2 ± 0.96 ^b^
31.25	0.072	0.069	0.066	0.069 ± 0.003	74.1 ± 3.06 ^c^
62.5	0.053	0.055	0.059	0.056 ± 0.003	59.8 ± 3.25 ^d^
125	0.043	0.049	0.048	0.047 ± 0.003	50.1 ± 3.31 ^e^
250	0.030	0.029	0.033	0.031 ± 0.002	32.95 ± 1.70 ^f^

^a^ Untreated control cells; ^b^ 250 µg/mL; ^c^ 125 µg/mL; ^d^ 62.5 µg/mL; ^e^ 31.25 µg/mL; ^f^ 15.62 µg/mL.

## Data Availability

The original contributions presented in the study are included in the article; further inquiries can be directed to the corresponding author.

## Abbreviations

The following abbreviations are used in this manuscript:

β	Peak broadening (in radians)
CO_2_	Carbon dioxide
CuNPs	Copper nanoparticles
DMEM	Dulbecco’s Modified Eagle’s Medium
DMSO	Dimethyl sulfoxide
DPPH	2,2-Diphenyl-1-picrylhydrazyl
EDAX/EDS	Energy dispersive X-ray analysis/Energy dispersive X-ray spectroscopy
*E. coli*	*Escherichia coli*
FESEM	Field emission scanning electron microscopy
FTIR	Fourier transform infrared spectroscopy
FWHM	Full width at half maximum
GC–MS	Gas chromatography–mass spectrometry
hkl	Miller indices
IC_50_	Half maximal inhibitory concentration
SPR	Surface plasmon resonance
